# Kindlin-2 is required for myocyte elongation and is essential for myogenesis

**DOI:** 10.1186/1471-2121-9-36

**Published:** 2008-07-08

**Authors:** James J Dowling, Andrew P Vreede, Susie Kim, Jeffrey Golden, Eva L Feldman

**Affiliations:** 1 Department of Pediatrics, University of Michigan, Ann Arbor, USA; 2 Department of Neurology, University of Michigan, Ann Arbor, USA; 3 Department of Neuropathology, Children's Hospital of Philadelphia, Philadelphia, USA

## Abstract

**Background:**

Integrins are required for normal muscle differentiation and disruptions in integrin signaling result in human muscle disease. The intracellular components that regulate integrin function during myogenesis are poorly understood. Unc-112 is an integrin-associated protein required for muscle development in C. elegans. To better understand the intracellular effectors of integrin signaling in muscle, we examined the mammalian homolog of Unc-112, kindlin-2.

**Results:**

Kindlin-2 expression is upregulated during differentiation and highly enriched at sites of integrin localization. RNAi knockdown of kindlin-2 in C2C12 cells results in significant abnormalities during the early stages of myogenesis. Specifically, differentiating myocytes lacking kindlin-2 are unable to elongate and fail to fuse into multinucleated myotubes. These changes are correlated with decreased cell substratum adhesion and increased cell motility. They are also associated with redistribution of a known kindlin-2 binding partner, integrin linked kinase (ILK), to the membrane insoluble subcellular fraction.

**Conclusion:**

In all, our study reveals kindlin-2 as a novel integrin adaptor protein important for muscle differentiation, and identifies it particularly as a critical regulator of myocyte elongation.

## Background

Skeletal muscle is a highly specialized tissue composed of multinucleated myotubes. The formation of myotubes from mononucleated precursor myoblasts via myogenesis is a complex, highly regulated process requiring both genetic and morphologic changes[1,2]. Understanding the molecular components of myogenesis is of high biologic and clinical importance, as the differentiation program is employed not only during development but also in the muscle regeneration response to injury[3]. In addition, several myopathies in humans are the result of aberrant muscle differentiation[4].

Integrin receptor mediated adhesion and signaling is required for proper myogenesis[5]. Specifically, integrin function has been demonstrated both *in vitro *and *in vivo *to be required for myoblast fusion and for sarcomerogenesis[6,7]. In addition, integrins are implicated in many other aspects of myogenesis, especially those related to the morphologic changes that accompany the differentiation process. The importance of integrins in skeletal muscle is underscored by the association between integrin abnormalities (both direct and indirect) and the pathogenesis of muscular dystrophy[8,9].

Numerous cytosolic effector molecules localize to integrin junctions and are associated with fundamental integrin-regulated processes like cell adhesion and cell migration[10]. However, the specific effectors that are responsible during myogenesis for transducing integrin signals, and thus for regulating integrin function in muscle, are not well established. Exceptions are integrin-linked kinase (ILK), which likely has multiple important roles[11,12], filamin-C, which is important for myoblast fusion[13], and focal adhesion kinase (FAK), which participates in the regulation of costamerogenesis[14].

We are interested in identifying other integrin-associated cytoplasmic adaptors important for myogenesis. One interesting potential candidate is kindlin-2/mig-2, a FERM and PH domain-containing protein that localizes to focal contacts and directly binds to the tail domain of β1 integrin[15,16]. Kindlin-2 is a member of the kindlin family of proteins[17]. These proteins are so named because of the association between kindlin-1 mutations and Kindler Syndrome, an epidermal blistering/skin fragility syndrome[18]. The kindlin family represents the vertebrate homologs of unc-112, a C. elegans protein implicated in integrin signal transduction during myogenesis[19]. Unc-112 specifically functions in the establishment of the linkage between the nematode integrins and the sarcomeric protein complex. Loss of unc-112 results in severe abnormalities in the organization and stability of the C. elegans body wall musculature.

Because of the important role of unc-112 in regulating integrin dependent aspects of muscle development in C. elegans, it is likely that kindlins are critical for vertebrate myogenesis. Kindlin-2 function is of particular interest as it is the only unc-112 homolog appreciably expressed in muscle *in vivo*[17,20]. Our previous work has revealed an essential role for kindlin-2 in vertebrate development[20]. Gene targeting in the mouse results in early embryonic lethality, and gene knockdown in the zebrafish causes abnormalities in multiple organ systems. Kindlin-2 loss in the zebrafish produces a severe cardiac and skeletal myopathy, and demonstrates a specific requirement for kindlin-2 in mediating the attachment of myofibrils to the sarcolemmal membrane.

In the present study, we focused on the role of kindlin-2 during the early stages of myogenesis. To test kindlin-2's function, we utilized the well-characterized C2C12 cell culture model system. C2C12 cells, when induced by serum withdrawal, go through a series of developmental stages that eventually produce a multinucleated myotube capable of spontaneous contraction. We first defined kindlin-2 expression during C2C12 myogenesis, and found it upregulated during the differentiation stage just prior to myoblast fusion and characterized by myocyte elongation. At these time points the protein is localized to sites of integrin expression. Using RNAi mediated gene knockdown, we then examined the function of kindlin-2. Kindlin-2 was required for differentiation to proceed through the stage of myoblast fusion. In particular, cells with reduced levels of kindlin-2 failed to spread and expand during the developmental phase of myocyte elongation. The myocytes exhibit decreased adhesion to fibronectin and instead had an increased capacity for migration. They were also characterized by an abnormal distribution of integrins and integrin associated proteins. In particular, the integrin associated protein, integrin linked kinase (ILK) was redistributed to the membrane insoluble fraction. In all, our study reveals that the integrin associated cytoplasmic effector molecule kindlin-2 is a critical regulator of myogenesis, and implicates kindlin-2 and integrin junctions in the regulation of myocyte elongation. Further, we propose that kindlin-2 functions by stabilizing and promoting cell-cell adhesion over cell migration.

## Results

### Kindlin-2 is enriched during early myogenesis

To study the expression and function of kindlin-2 during myogenesis, we utilized the well-characterized C2C12 cell culture model system. Proliferating C2C12 cells resemble fibroblasts, but upon serum removal they withdraw from the cell cycle, elongate, and fuse to form multinucleated myotubes. These stages of differentiation are accomplished within the first four days after serum withdrawal, with myoblast fusion usually beginning by day 3 or 4.

**Figure 1:**
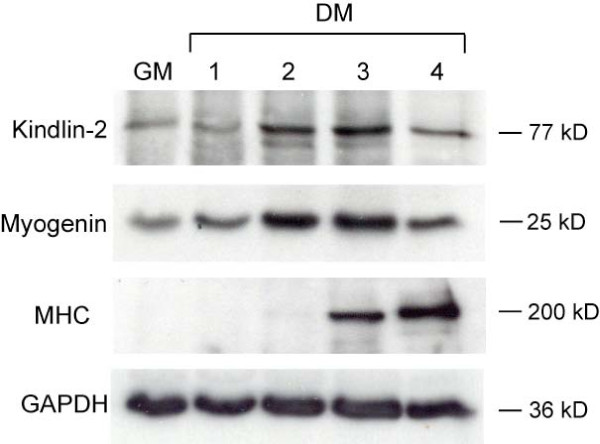
**Kindlin-2 expression during myogenesis *in vitro***. Western analysis of kindlin-2 on C2C12 cell protein extracts. C2C12 cells we cultured in either growth media (GM) or differentiation media (DM) for 1, 2, 3, or 4 days. Kindlin-2 levels were compared to an early (myogenin) and a late (myosin heavy chain; MHC) marker of differentiation. GAPDH was used as a loading control. Kindlin-2 expression was induced upon differentiation, with a peak expression at day 3 post-differentiation. Of note, the GM cell extract was from confluent cells, and confluence stimulates a small amount of differentiation.

We examined kindlin-2 expression using Western blot analysis in confluent, proliferating C2C12 cells and during the first four days after serum withdrawal-induced myogenesis (Figure 1). In undifferentiated cells (GM), kindlin-2 is present at low levels. Upon induction of differentiation (DM), kindlin-2 levels rise, peaking at day 3. At DM3, kindlin-2 expression is approximately 5-fold higher than in undifferentiated cells. We compared kindlin-2 levels with those of the differentiation markers myogenin (myog) and myosin heavy chain (MHC). Myogenin is induced upon cell cycle withdrawal, while MHC is expressed coincident with the onset of myoblast fusion. Kindlin-2 levels rise after myogenin but before MHC, at time points consistent with the myocyte elongation stage of differentiation.

### Kindlin-2 localizes to focal contacts and developing costameres

**Figure 2:**
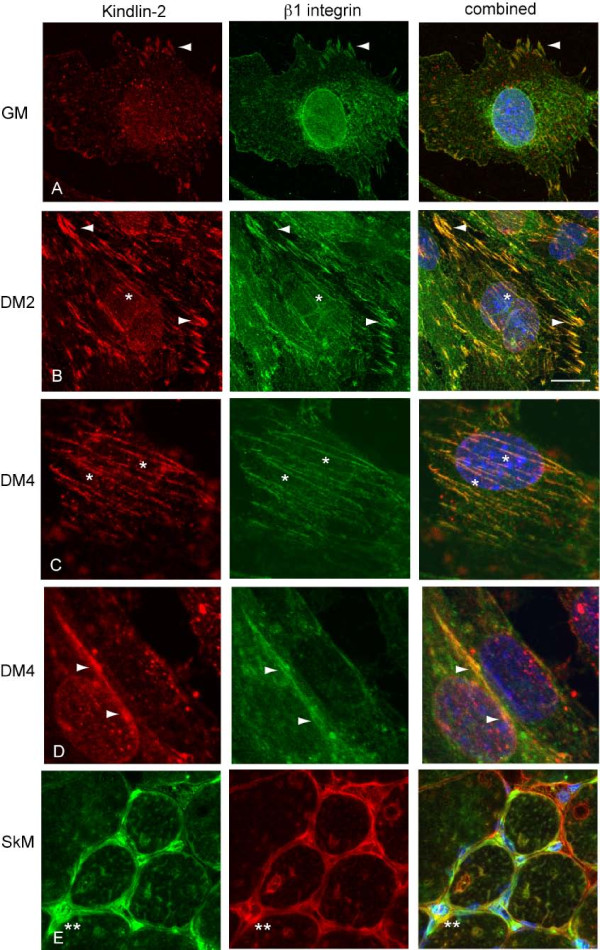
**Kindlin-2 subcellular localization**. A.-D. Confocal micrographs of immunohistochemistry using anti-kindlin-2 and anti-β1 integrin antibodies on undifferentiated (A; GM) C2C12 cells and C2C12 cells induced to differentiate for 2 (B; DM2) or 4 days (C, D; DM4). Kindlin-2 co-localizes with β1 integrin at sites of cell-cell and cell-matrix contacts (arrowheads in A, B, and D). Kindlin-2 is found at developing costameres at DM2 and DM4 (*). Scale bar = 20 μm. E. Immunostaining of quadriceps muscle from a two month-old mouse. Kindlin-2 localizes to the sarcolemmal membrane and is enriched in the perinuclear compartment (**).

We next determined the subcellular localization of kindlin-2 during myogenesis using immunohistochemistry. In undifferentiated C2C12 cells, kindlin-2 expression is faint but detectable at membrane protrusions and cell-matrix contacts (Figure 2A; arrowheads). It co-localizes with β1 integrin in a pattern consistent with expression at focal contacts. The staining in undifferentiated cells is similar to that reported for kindlin-2 in non-myogenic cell lines.

Upon differentiation, kindlin-2 immunofluorescence noticeably increases, in agreement with our Western blot data. In two day differentiated C2C12 cells, kindlin-2 is highly enriched in puncta along the plasma membrane (arrowheads, Figure 2B). These puncta also contain β1 integrin, and likely represent areas of cell-cell and cell-matrix adhesion. In addition, staining is observed along linear structures within the cell (*). In four day differentiated cells, kindlin-2 is primarily expressed in this linear staining pattern (Figure 2C). At this structure, it co-localizes with β1 integrin and focal adhesion kinase (FAK) (Figure 2B–C and data not shown). As corroborated by previously published reports of β1 integrin and FAK expression patterns[14], this kindlin-2 staining corresponds to the developing costamere. Costameres are integrin-containing junctions that link sarcomeres to the plasma membrane[21]. They are believed to represent the differentiated muscle equivalent of the focal contact. One additional site of kindlin-2 localization is along the plasma membrane between adjacent cells (Figure 2D). As with its expression at other subcellular compartments, it colocalizes with β1 integrin at these cell-cell contacts.

To support the relevance of the abundant staining of kindlin-2 in C2C12 cells, we examined expression in mouse skeletal muscle (Figure 2E). As with cell culture, anti-kindlin-2 strongly stains the sarcolemmal membrane in muscle cross sections and overlaps with the expression of β1 integrin. Kindlin-2 also appears enriched in the perinuclear area (**), an organelle rich domain that is enriched with neuromuscular junctions.

### RNAi knockdown of kindlin-2 alters myogenesis

**Figure 3:**
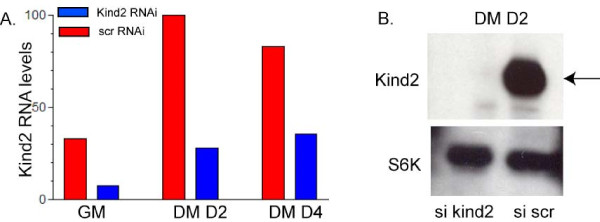
**RNAi reduces kindlin-2 levels**. A. Quantitative PCR analysis of C2C12 cells treated with kindlin-2 or scrambled siRNA. Samples for analysis were collected 1 day (GM), 3 days (DM day 2) and 5 days (DM day 4) after transfection. Relative kindlin-2 levels were (si scrambled vs. si mig2): 33% vs. 8% (GM), 100% vs. 28% (DM day 2), and 83% vs. 36% (DM day 4). Numbers reflect percent level as compared to scrambled RNAi at DM day 2. Values were averaged from 4 independent transfections, each analyzed in triplicate. B. Immunoblot of kindlin-2 on protein extracts obtained after 2 days in DM (72 hrs after transfection). A band corresponding to kindlin-2 (arrow) is observed in the scrambled RNAi lane only. Equal loading was confirmed by reprobing the blot with S6 kinase (S6K) and GAPDH (data not shown).

**Figure 6:**
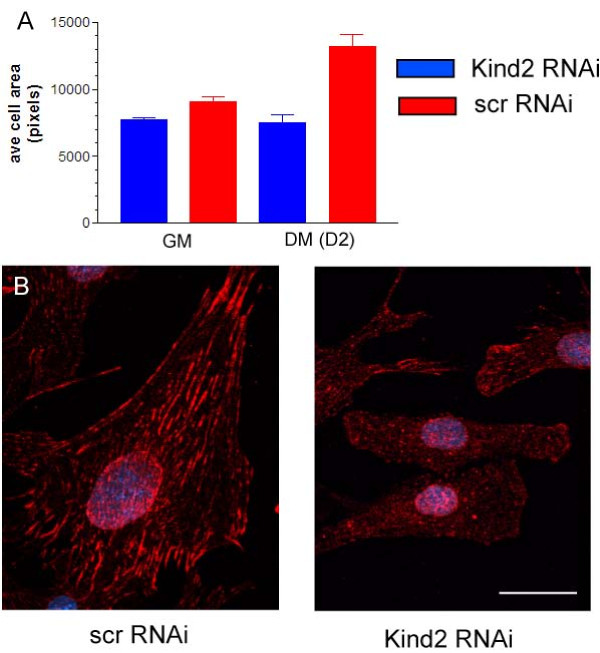
**Myocyte elongation is impaired by kindlin-2 knockdown**. A. C2C12 cells were transfected with RNAi and either maintained in Growth Media (GM) or changed to Differentiation Media for two days (DM D2). Cellular area was determined using Metamorph on immunostained cells overexposed to reveal their cell borders. There was a small but significant difference between conditions in undifferentiated cells (kindlin-2 RNAi vs. scrambled RNAi: 7692 +/- 172 pixels v 9080 +/- 348 pixels, n= 240 cells, p = 0.006). There was a substantial difference between conditions in differentiated cells (kindlin-2 RNAi vs. scrambled RNAi: 7510 +/- 599 pixels v 13180 +/- 906 pixels, n = 270, p = 0.001). B. C2C12 cells at differentiation day 2 immunostained with anti-kindlin-2. The photomicrographs emphasize the obvious size difference between scrambled RNAi transfected cells and those transfected with kindlin-2 RNAi. They also reinforce the successful knockdown of kindlin-2 protein by RNAi. (scale bar = 20 μm).

To study the function of kindlin-2 during myogenesis, we designed synthetic RNAi to the murine kindlin-2 gene. The extent of knockdown was determined using quantitative RT-PCR (qPCR) (Figure 3A), Western blot analysis (Figure 3B), and immunohistochemistry (see Figure 6B). Based on the qPCR data, we consistently observed 70–80% knockdown 24 hours after RNAi transfection. This effect was maintained at 3 days after transfection (differentiation day 2), but diminished to approximately 50% by 5 days (differentiation day 4). The specificity of the RNAi for kindlin-2 knockdown and the observed phenotypic changes were verified using an independent RNAi designed to a second part of the gene. Information for each RNAi is listed in the materials and methods. Of note is that the homologous gene products kindlin-1 and kindlin-3, as determined by RT-PCR, are not expressed in C2C12 cells under control conditions and are not upregulated with kindlin-2 knockdown.

**Figure 4:**
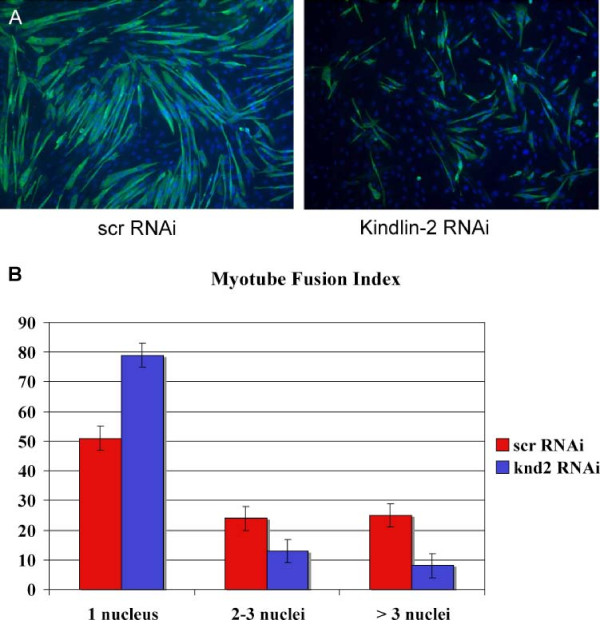
**Myoblast fusion and kindlin-2 knockdown**. A. C2C12 cells transfected with scrambled (scr) or kindlin-2 RNAi, incubated in differentiation media for 4 days, and then immunostained with anti-myosin heavy chain. Note that most MHC positive myoblasts treated with kindlin-2 RNAi do not form elongated, multinucleated myotubes. B. Myotube Fusion Index (MFI). The MFI was calculated using immunostained cells as in (A). The number of nuclei (labeled with DAPI) were counted in each MHC positive cell. There were significantly fewer fused myotubes in kindlin-2 knockdown cells. Results were (scrambled vs. kindlin-2): 1 nuclei 51.1+/-2.9% vs. 75.6+/-2.6% (p = 0.0001); 2–3 nuclei 25.5+/-2.6% vs. 15.4+/-1.0% (p = 0.0036); >3 nuclei 23.4+/-1.3% vs. 8.9+/-1.6% (p < 0.0001). Counts were obtained from 5 independent trials/transfections, and at least 250 cells from each condition were counted per trial.

We began our analysis of kindlin-2 function by determining the ability of C2C12s with reduced kindlin-2 levels to differentiate through the stage of myoblast fusion. We chose this differentiation end point because it typically occurs 3–4 days after serum withdrawal, and kindlin-2 levels are highest in the days just prior to it. To measure myoblast fusion, we calculated the myoblast fusion index (MFI). The MFI is determined by immunostaining day 4 differentiated cells (DM4) with myosin heavy chain (MHC), counting the number of nuclei in each MHC positive cell, and then comparing the number of multinucleated cells to the overall number of MHC positive cells. Myotubes were considered partially fused if they contained 2–3 nuclei, and fully fused if they contained 4 or more nuclei. In C2C12 cells transfected with a scrambled RNAi, 25.5% were partially fused and 23.4% were fully fused (Figure 4A, B). These data are consistent with previously published reports on myotube fusion at DM4[22]. In contrast, in C2C12 cells transfected with kindlin-2 RNAi, only 15.4% were partially fused (vs. 25.5%, p = 0.0036) and only 8.9% fully fused (vs. 23.4%, p < 0.0001) (Figure 4A, B). Overall, while approximately half of all control/scrambled cells fused, only 25% of cells from kindlin-2 RNAi transfections were able to differentiate to the point of even partial fusion, suggesting a delay or arrest in differentiation prior to or at the stage of myoblast fusion.

**Figure 5:**
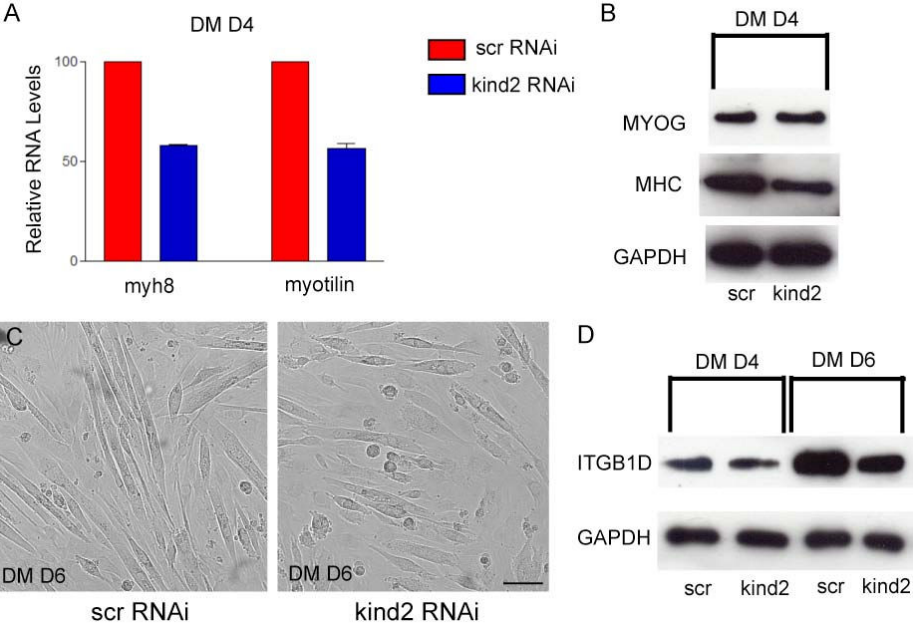
**Altered expression of differentiation markers and kindlin-2 knockdown**. A. Relative RNA levels of myh8 and myotilin in RNAi transfected, 4 day differentiated C2C12 cells as determined by qPCR. Each value was calculated from 3 independent transfections with qPCR performed in triplicate. In kindlin-2 RNAi transfected cells, myh8 and myotilin levels were 58 +/-1% and 56+/-3%, respectively, of the values obtained for control cells. B. Immunoblot on protein extracted from day 4 differentiated samples transfected with scrambled or kindlin-2 RNAi. Blots were probed with the following antibodies: myogenin (myog), myosin heavy chain (MHC), and GAPDH (loading control). Densitometric analysis revealed no difference in myogenin levels between samples, and a reduction to 75% of normal for MHC. C. Brightfield images of scrambled (scr) or kindlin-2 (kind2) RNAi transfected C2C12 cells incubated in differentiation media for 6 days (DM D6). Note extensive myotube formation in the scrambled sample as compared to the kindlin-2 RNAi sample. D. Immunoblot of day 4 (DM D4) and day 6 (DM D6) differentiated myocytes transfected with scrambled (scr) or kindlin-2 (kind2) RNAi. Blots were probed with antibodies to β1D integrin and GAPDH. Densitometric analysis revealed 75% and 77% reductions respectively in β1D integrin levels in kindlin-2 RNAi samples.

To confirm that kindlin-2 RNAi significantly hindered myogenesis, we measured the levels of various markers of differentiation using both qPCR and Western blot analysis. We examined myosin heavy chain isoform 8 (myh8) and myotilin RNA levels at differentiation day 4 using qPCR. Both genes are expressed starting at the stage of myoblast fusion. As expected from our myotube fusion data, both myh8 and myotilin levels were substantially lower in kindlin-2 RNAi transfected cells as compared to scrambled RNAi transfected cells (Figure 5A). We utilized Western analysis of myosin heavy chain to substantiate our RNA data. As shown in Figure 5B, MHC protein levels are also diminished at differentiation day 4. Myogenin protein levels were also examined, and were undistinguishable between scrambled and kindlin-2 RNAi samples. This suggests that differentiation proceeds through the point of myogenin induction, but is altered prior to later events like fusion.

To address if differentiation is simply delayed as opposed to arrested, we examined cultures incubated for 6 days in differentiation media. At this time point, because our RNAi is delivered via transient transfection, kindlin-2 levels are recovered to greater than 50% of normal (data not shown). Despite this, there continues to be a reduction in the number of fused myotubes (Figure 5C). In addition, as determined by Western blot analysis, there are reduced levels of the late differentiation marker β1D integrin (Figure 5D). These data suggest an arrest of myogenesis prior to the stage of myoblast fusion.

### Kindlin-2 knockdown prevents myoblast elongation

A key event during *in vitro *differentiation is myocyte elongation[22]. Elongation directly precedes myoblast fusion. Given that integrin adhesion is implicated in the elongation process, we next examined the extent to which RNAi transfected cells increased in cellular volume after differentiation. We accomplished this by measuring the two-dimensional attached area of cells just prior to differentiation and then two days after induction of differentiation. Undifferentiated C2C12 cells transfected with kindlin-2 RNAi, while phenotypically normal in appearance, had slightly less attached area than controls (7,000 pixels vs. 8,000 pixels; Figure 6A). Upon differentiation, however, there was a dramatic difference in calculated areas. Cells transfected with scrambled RNAi increased to 14,000 pixels by differentiation day 2, while kindlin-2 RNAi treated cells only increased to 8,000 pixels (Figure 6A). This statistical difference was also apparent by visual inspection and on cells immunostained with various antibodies. One example is depicted in Figure 6B, which shows D2 differentiated cells stained with anti-kindlin-2. As compared to cells transfected with scrambled siRNA (left panel), the cells with reduced levels of kindlin-2 were clearly smaller and had a non-extended morphology (right panel). In all, these data indicated that reduction of kindlin-2 levels resulted in a failure of myocyte elongation.

### Kindlin-2 knockdown alters cell spreading and cell migration

To better understand the reason myocytes with reduced kindlin-2 levels do not properly elongate, we measured their ability to spread/adhere to different extracellular matrices and also to migrate through transwell chambers. Cells were transfected with RNAi, differentiated for 2 days by serum withdrawal, and then trypsinized in preparation for the relevant assays.

**Figure 7:**
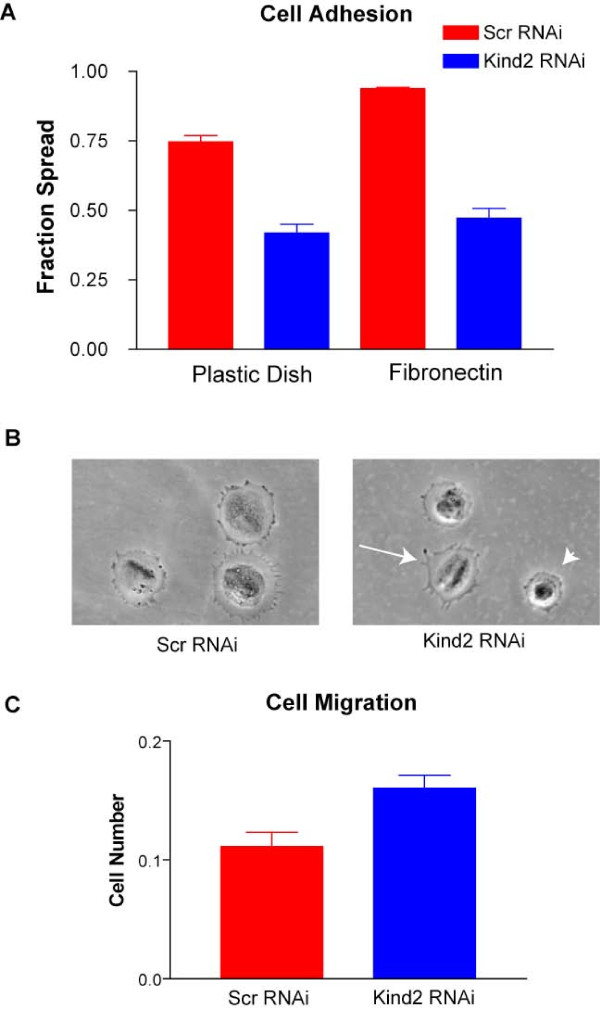
**Kindlin-2 knockdown alters C2C12 adhesion and migration**. A. Day 2 differentiated myocytes were plated onto either non coated or fibronectin coated cell culture dishes and incubated for 1 hour at 370C. Cells were fixed and then examined for morphologic changes consistent with adhesion and spreading. On non coated dishes, percent spreading was 71.6% (+/-0.02%) for scrambled and 41.8% (+/-0.03%) for kindlin-2 RNAi (p < 0.0001, n = 5). On fibronectin, percent spreading was 93. 8% (+/-0.01%) for scrambled and 47.2% (+/-0.03) for kindlin-2 RNAi (P < 0.0001, n = 5). B. Bright field photomicrograph taken at 10× of representative cells from the assay described in A. The arrow points to a spread cell while the arrowhead points to a non-spread cell in kindlin-2 RNAi transfectents. C. RNAi transfected C2C12 cells were replated onto transwell and allowed to migrate through the wells to a lower chamber filled with media. Migrated cells were counted after 12 hours. Significantly more migration was observed with kindlin-2 knockdown (scrambled v kindlin-2 RNAi, 0.11+/-0.01 v 0.16+/-0.01; p = 0.019; n = 3).

To study cell adhesion, cells were replated onto plastic, fibronectin, laminin-1 and collagen IV. After replating for one hour onto plastic dishes, 75% of control cells adhered and spread. Conversely, only 42% of kindlin-2 RNAi cells spread (Figure 7A, p < 0.0001, n = 5 trials). A similar decrease in spreading was observed with laminin and collagen IV coated dishes (data not shown). Replating on fibronectin, however, produced a much more dramatic difference. At one hour, 94% of control cells spread on fibronectin dishes, an expected increase over plastic dishes given that α5β1, a fibronectin receptor, is the predominant integrin heterodimer expressed at differentiation day 2[23,24] (Figure 7A). On the other hand, only 47% of kindlin-2 RNAi transfectants spread on this surface, a value only marginally higher than that recorded for plastic dishes (Figure 7A, p < 0.0001, n = 5 trials). Representative RNAi transfected cells replated onto fibronectin are shown in Figure 7B.

We then examined whether cell motility was also affected by kindlin-2 knockdown. To test migration, siRNA transfected cells were replated onto the upper surface of transwell chambers. Using crystal violet staining as an indicator of cell number, we measured the number of cells which migrated through to the bottom of the transwell after 24 hours. We observed a substantial difference between conditions, with significantly more cell migration detected in chambers with cells exposed to kindlin-2 RNAi (Figure 7C). This finding demonstrates that loss of kindlin-2 has a pro-migratory effect on C2C12 cells. When considered with our observation that kindlin-2 deficient myoblasts fail to properly elongate and have altered substratum adhesion, it suggests that kindlin-2 is critical for the transition of C2C12 cells from a motile state to a strongly adhered, elongated state.

Given these changes in cell adhesion and cell motility, and in particular the changes observed with spreading on fibronectin, we examined the possibility that kindlin-2 knockdown affected extracellular matrix protein or integrin receptor expression. Using a combination of RT-PCR, real time PCR, and Western blot analysis, we detected no difference in the RNA or protein levels of fibronectin, laminin and merosin, and no alteration of RNA levels of α5 or α7 integrins (data not shown).

### Kindlin-2 knockdown alters ILK localization

**Figure 8:**
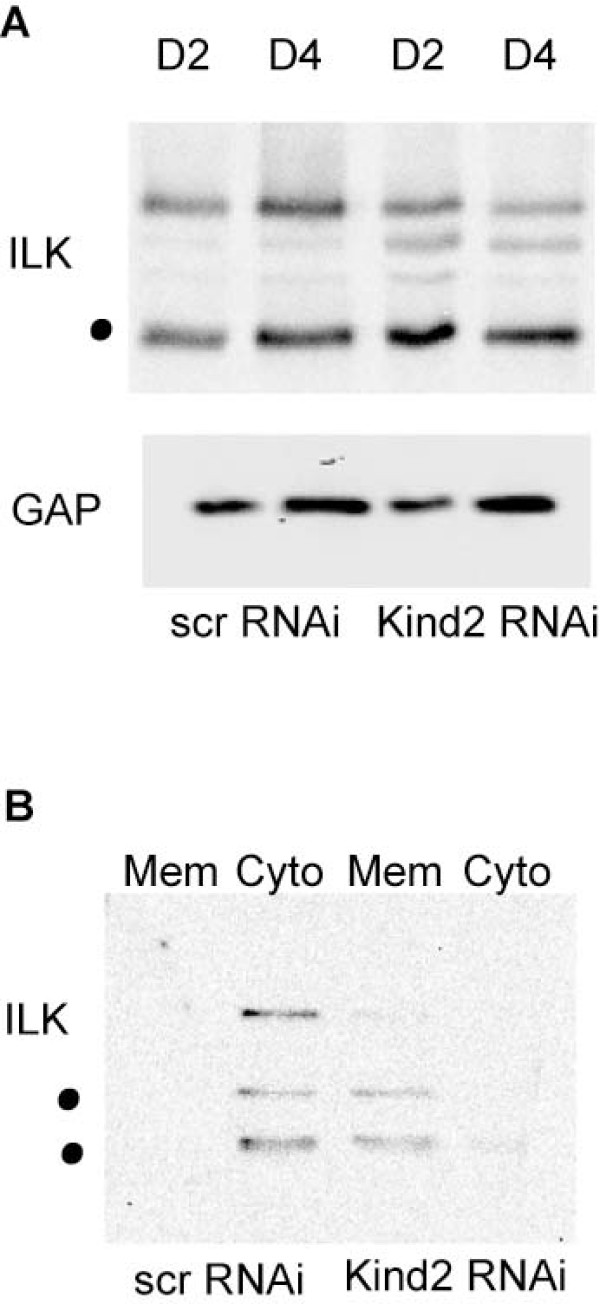
**ILK localization is altered with kindlin-2 knockdown**. A. Immunoblot on protein from C2C12 cells at differentiation day 2 (D2) and day 4 (D4) transfected with scrambled (scr) or kindlin-2 (kind2) RNAi. Blots were probed with anti-ILK and anti-GAPDH. No difference by densitometry was detected between scrambled and kindlin-2 RNAi cells. Dot indicates ILK band. B. Immunoblot on subcellular fractions from day 2 differentiated C2C12 cells probed with anti-ILK. ILK localizes to the cytosolic (cyto) fraction in scrambled RNAi cells and to the membrane/insoluble (mem) fraction in kindlin-2 RNAi cells. No expression was seen with either condition in the cytoskeletal or nuclear fractions (data not shown).

In C. elegans, unc-112, the worm homolog of kindlin-2, binds to and modulates the function of Pat-4, the worm homolog of ILK[25]. Because ILK is required for mammalian myogenesis both *in vivo *and *in vitro*, we wanted to establish the effect of kindlin-2 knockdown on ILK expression and localization. At differentiation days 2 and 4, the overall levels of ILK were unchanged between conditions (Figure 8A), yet the localization of ILK was significantly altered. As determined using subcellular fractionation, essentially all ILK in control transfected cells was present in the cytosolic fraction at differentiation day 2 (Figure 8B). This is consistent with previously published reports on ILK localization in C2C12 cells[12]. In contrast, ILK was found only in the membrane/insoluble fraction in the kindlin-2 knockdown myocytes (Figure 8B). Thus, in the absence of kindlin-2, ILK fails to redistribute to its proper cytosolic localization during myogenesis.

### Kindlin-2 knockdown affects focal contact distribution

**Figure 9:**
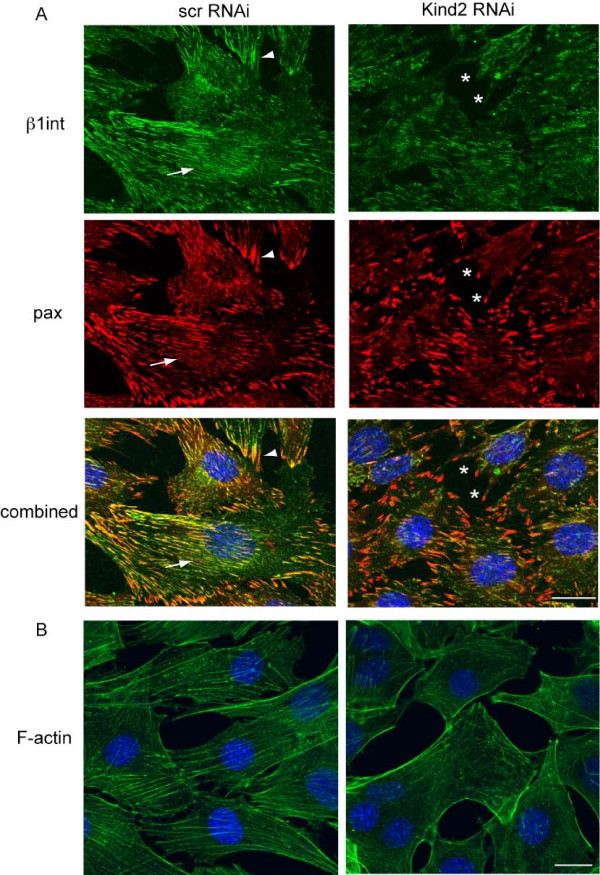
**Kindlin-2 knockdown and focal adhesion localization**. A. Confocal immunofluorescent microscopy with anti-β1 integrin (green) and anti-paxillin (red) on C2C12 cells transfected with RNAi and then changed to differentiation media for 2 days. Control cells (scr RNAi) show linear staining consistent with localization to costameres (arrows), as well as punctate focal contact staining (arrowheads). Conversely, focal contact proteins in the kindlin-2 RNAi cells fail to form linear structures and instead are concentrated in unusual appearing puncta (*). (Scale bar = 20 μM). B. Oregon Green phalloidin staining on day 2 differentiated C2C12 cells. In the scrambled RNAi myocytes, actin is found primarily in a linear, fiber like pattern. Conversely, in the kindlin-2 RNAi cells it is found concentrated at the membrane. (Scale bar = 20 μM).

We next examined other focal contact components for similar changes in subcellular localization. In control transfected cells at differentiation day 2, we observed the expected pattern of expression for β1 integrin and paxillin both in discrete points along the membrane as well as in linear structures throughout the cells (Figure 9A). In kindlin-2 RNAi transfected cells, we detected a relative shift in this distribution, with concentration of the staining in punctate at the sarcolemmal membrane. Of note is that we did not see obvious changes in the subcellular localization of FAK or migfilin (data not shown).

**Figure 10:**
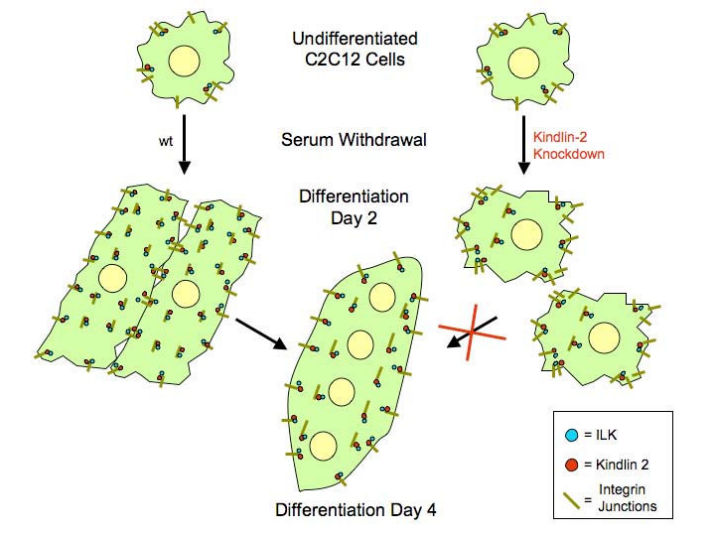
**Schematic of kindlin-2 function during early myogenesis**. A. Undifferentiated C2C12 cells with typical fibroblast-like morphology B. Upon change to differentiation media, control cells withdraw from the cell cycle and elongate. In cells with reduced levels of kindlin-2, cells fail to elongate, and instead maintain a "pro-migratory" phenotype. C. By differentiation day 4, elongated control cells fuse into multinucleated myotubes. Reduced levels of kindlin-2 result in failure of myotube formation, likely as a result of decreased elongation, inadequate cell adhesion and impaired myoblast fusion. One mechanism underlying these alterations is a failure of redistribution of ILK containing focal adhesions.

We also measured F-actin staining using Oregon Green conjugated phalloidin. We again observed a qualitative redistribution of F-actin from a linear pattern in control myocytes to concentration along the sarcolemmal membrane in kindlin-2 RNAi myocytes (Figure 9B). Taken together with our ILK results, these data suggest that integrin based, ILK-containing adherens junctions are inappropriately distributed in differentiating myocytes. We hypothesize that, in the absence of kindlin-2, ILK enriched focal adhesions fail to redistribute into an extensive linear pattern during myogenesis, and that this in turn results in defective myocyte elongation (Figure 10).

## Discussion

C2C12 cells undergo a well-documented series of steps after induction of differentiation by serum withdrawal[22]. Within 24 hours, cells exit the cell cycle and begin expressing muscle specific transcription factors. The next stage is myocyte elongation, where dramatic changes in cell morphology occur between 1 and 3 days after induction. Elongation is followed by myoblast fusion, which begins at differentiation day 3. In this study, we describe for the first time the role of kindlin-2 in these early stages of differentiation. In particular, we identify kindlin-2 as a novel integrin associated cytoplasmic adaptor protein important for the process of myocyte elongation.

Myocyte elongation, while a well-described morphologic phenomenon, is not well characterized at the molecular level. As revealed by two published studies, integrin signaling has been previously implicated in the process. In the first, alteration of integrin binding to the extracellular matrix by manipulation of matrix metalloprotease function in C2C12 cells causes abnormalities in myocyte elongation[22]. In the second study, overexpression of the focal contact associated protein FHL1/SLIM induces hyper-elongation, with the hyper-elongation dependent on integrin-ligand binding[26]. Our study clearly supports and advances this association between integrin junctions and myocyte elongation. Furthermore, it introduces kindlin-2 as one of the key molecular regulators of the process.

How might kindlin-2 regulate elongation? Our hypothesis is that kindlin-2 promotes and stabilizes cell adhesions. This hypothesis is supported by several observations. The first is differentiating myocytes with reduced kindlin-2 expression have impaired spreading on all extracellular matrix surfaces and in particular on fibronectin. The second is that kindlin-2 expression appears to reduce the motile behavior of myocytes. This effect of cell migration has been previously demonstrated for kindlin-2 in a series of non-myogenic tumor cell lines[15]. Specifically, Wu and colleagues showed an inverse correlation between tumor cell motility and kindlin-2 levels, and additionally showed that expression of kindlin-2 in motile cell lines decreased their migration and strengthened their attachments. The third, as described in our previous study, is that loss of kindlin-2 in zebrafish muscle leads to less elaborate cell-cell junctions which, by ultrastructural analysis, do not have their typical associations with the underlying cytoskeleton[20]. In addition, we reported that both cardiac muscle and skeletal muscle in the fish are smaller in the absence of kindlin-2. The data from the present study suggest that this may be on the basis of failed myocyte elongation.

Understanding how precisely kindlin-2 stabilizes adhesion and promotes myocyte elongation is an open question deserving further study. Our data support a potential role for ILK in this process. Previous work on ILK, while clearly showing a requirement for the gene for both cardiac and skeletal myogenesis[11,12,27], has not specifically implicated the protein in myocyte elongation. However, ILK is associated both with the stabilization of adhesion and with the promotion of migration[28,29]. Participation in these seemingly paradoxical activities depends on ILK kinase activity, ILK localization and differential association with ILK binding partners. As known binding partners, kindlin-2 and ILK are logical candidates to share functions[25]. Similar to observations from C. elegans, we describe here altered localization of ILK with the reduction of kindlin-2 expression. Because ILK localization is a significant factor in whether it functions to promote adhesion or migration, a plausible mechanism for kindlin-2 function is that it localizes to integrin junctions in the differentiating myocyte and it in turn stabilizes ILK association with these junctions. This interaction then promotes adhesion and stimulates elongation, likely via actin cytoskeletal remodeling. Loss of kindlin-2 would then be expected to cause improper localization of ILK, potentially to sites that promote migration.

Regardless of the exact mechanism, the suggestion from our data that kindlin-2 is involved with ILK regulation is important in reference to human muscle disease. ILK has been both directly and indirectly implicated in cardiac hypertrophy[30], cardiomyopathy[31,32] and muscular dystrophy[11]. An association of kindlin-2 with ILK thus strengthens the hypothesis that kindlin-2 is likely to be important for human muscle diseases.

It is quite likely, however, that kindlin-2 function is transmitted in part by additional regulatory proteins. One intriguing molecule is FHL1, a LIM domain containing protein. Currently, other than β1 integrin, the only well-established binding partner for kindlin-2 is migfilin, which is also a LIM domain protein[16]. We did not observe alterations in migfilin expression, and migfilin has yet to be associated with skeletal myogenesis. Thus one possibility may be that kindlin-2 regulates elongation by recruiting FHL1 to integrin junctions. This hypothesis is of clinical relevance, as FHL1 mutations have been detected in patients with an X-linked muscle disease called Scapulo-Peroneal Syndrome[33,34]. Another possibility is that kindlin-2 facilitates and stabilizes the association between integrin junctions and the underlying actin cytoskeleton. Such a role is supported by the function of its C. elegans homolog unc-112 and by our previous published data from the zebrafish[19,20].

An additional event in myogenesis that may require kindlin-2 function is myoblast fusion. Integrin junctions have a well established association with fusion[7], and kindlin-2 is expressed at the correct development time (3–4 days after induction of differentiation) and in the correct location (co-localized with integrin at sites of cell-cell attachment). We clearly observe a defect in the ability of kindlin-2 RNAi transfected cells to proceed through the stage of myoblast fusion. Alternatively, this observation may be explained by the fact that these myocytes have defective elongation, and elongation may be required for fusion to proceed. Further experimentation is thus necessary to resolve this potential function.

## Conclusion

In summary, we have demonstrated that kindlin-2 is a critical regulator of early myogenesis *in vitro*. Its specific functions in myocyte elongation suggest it is a novel regulator of integrin signaling during myogenesis. Based on its expression and function, kindlin-2 represents a potentially important factor in integrin-related human muscle pathologies, especially the muscular dystrophies.

## Methods

### Tissue culture

C2C12 cells were obtained from ATCC. They were maintained in growth media (GM; DMEM (Gibco) + 10% FBS (HyClone)) and differentiated in differentiation media (DM; DMEM + 2% Horse Serum (HyClone)). Cells were grown on 60 mm plastic ware (Falcon) or on 22 mm coverglass coated with Fibronectin (BD Biosciences). All transfections were done with Lipofectamine 2000 (Invitrogen). RNAi specific for mouse Kindlin-2 was obtained from Invitrogen (oligo IDs MSS211904/5). Stealth Negative Control Medium was used as the scrambled control (Invitrogen). Transfection efficiency of the RNAi was > 90%, as determined by co-transfection with the BLOCK-iT reagent (FITC conjugated; Invitrogen).

### Immunofluorescence

Immunofluorescence microscopy was performed on C2C12 cells and cryosections from fresh frozen adult mouse quadriceps using standard methodology. Cells were fixed for 10 minutes at 4°C in 1:1 methanol:acetone or at room temperature in 4% paraformaldehyde and tissue sections were fixed for 5 minutes at room temperature in acetone. The following primary antibodies were used: goat anti-Kindlin-2 (1:25; Santa Cruz); mouse anti-kindlin-2 (1:30; Abnova); mouse anti-paxillin (1:300; Transduction Laboratories); anti-β1 integrin-FITC (1:100; Santa Cruz); rat anti-β1 integrin (1:25; Chemicon); mouse anti-myosin heavy chain (MF20; 1:30; Developmental Hybridoma Bank), and Oregon Green phalloidin (1:200; Invitrogen). Alexafluor-conjugated secondary antibodies were used at 1:250 (Invitrogen). Nuclei were counterstained with ProLong Gold plus DAPI (Invitrogen). Analysis was performed with an Olympus FluoView 500 laser scanning confocal microscope. Samples were scanned on an Olympus IX-71 inverted confocal microscope using either a 60× or 100× oil immersion objective and captured using the FluoView v4.3 software.

### Immunoblots

Protein was extracted from C2C12 cell cultures using RIPA buffer with Complete Protease Inhibitors (Roche). Samples were quantitated using the Bradford assay (Pierce). 40 μg or 70 μg (for kindlin-2) of extract was resolved by SDS-PAGE, transferred to Immobilon (Millipore), and subjected to immunoblotting. Membranes were blocked for 1 hour with 5% milk in PBS containing 0.1% Tween. Membranes were incubated in primary and secondary antibodies for 1 hour at room temperature. Primary antibodies used were: mouse anti-kindlin-2 (1:250); mouse anti-GAPDH (1:1000); rabbit anti-myogenin (1:1000; abcam); mouse anti-MHC (1:1000); rabbit anti-ILK (1:1000; Millipore); rabbit anti-FAK (1:2000; Santa Cruz); rabbit anti-laminin (1:2000; Sigma); rabbit anti-fibronectin (1:200; Santa Cruz); β1D integrin (1:1000; Chemicon). Secondary antibodies were from Santa Cruz Biotechnology and used at 1:2000. Blots were developed using Lumiglo (Cell Signalling) and exposed to Hyperfilm (GE Biosciences). Protein extracts for cell fractionation studies were obtained using the Qproteome Cell Compartment kit (Qiagen). Successful fractionation was verified using the following antibodies: nuclei and membrane (insoluble)- emerin (1:1000; Abcam), cytoplasmic- GAPDH, cytoskeletal- actin (1:200, Santa Cruz).

### Real time PCR

RNA was extracted from C2C12 cell cultures using the Trizol reagent (Invitrogen). First strand cDNA was synthesized from 1 μg RNA using the Promega Reverse Transcription System with the Random Hexamer Primer. cDNA was diluted 1:10, and then 2 μL was used for Real Time PCR. Real Time PCR was performed using the QuantiTect SYBR-Green reagent and iCycler iQ PCR machine (Qiagen). Real Time primers were obtained from Qiagen. GAPDH was used as the amplification control.

### Cell size determination

Immunofluorescence using anti-β1 integrin FITC was performed on RNAi transfected C2C12 cells incubated in either growth media or differentiation media. Fluorescence was visualized at 20× using a Nikon Microphot Microscope. Digital images were obtained in MetaMorph using SPOT camera. Cell size was determined for mononuclear cells in MetaMorph with the free hand tool and area determination calculator. Cell areas were then analyzed and histograms generated using Prism. Single-tailed Student's t-test was used to determine statistical significance.

### Myoblast fusion index

Immunofluorescence using anti-MHC was performed on C2C12 cells at 4 days after differentiation. Nuclei were counterstained with DAPI. Digital images were captured at 20× as done above for cell size determination. The Myoblast Fusion Index was calculated as follows: MHC-positive cells were scored as having 1, 2–3, or >3 nuclei. Values were expressed as a percentage of the total number of MHC-positive cells counted. At least 250 cells were counted for each condition, and 4 separate trials were performed. Values were analyzed and histograms generated using Prism, and statistical significance determined using a single-tailed t-test.

### Myocyte adhesion assay

RNAi transfected C2C12 cells at differentiation day 2 were dissociated with trypsin and then replated onto 60 mM culture dishes. Dishes either had no coating or were precoated with laminin-1, collagen IV, or fibronectin (Bectin Dickinson). Cells were allowed to adhere for 60 minutes at 37°C in differentiation media, and then were fixed for 10 minutes at room temperature in 4% paraformaldehyde. Cells were examined with an inverted microscope at 20× magnification and scored as adhered if they assumed a spread morphology and non adhered/spread if they had a rounded morphology with no processes. 5 counts of at least 25 cells were performed for each condition.

### Myocyte migration assay

RNAi transfected C2C12 cells were dissociated with Dissociation solution (Sigma), counted with a hemocytometer, and plated on top of transwells (1 × 10^5 ^cells/well). Cells were incubated for 12 hours in DMEM/2%HS. Transwells (Costar) were then processed as follows: Cells were removed from inner wells with Q-tips. Outer wells were stained with crystal violet for 20 minutes, eluted with 10% acetic acid, and then measured on a plate reader at 590 nm.

## Authors' contributions

JJD carried out or supervised all experiments and drafted the manuscript. AV and SK performed certain experimentation. JJD, JG and ELF conceived of the study, participated in its design and edited the manuscript.

## References

[B1] Berkes CA, Tapscott SJ (2005). MyoD and the transcriptional control of myogenesis. Semin Cell Dev Biol.

[B2] Hollway GE, Currie PD (2003). Myotome meanderings. Cellular morphogenesis and the making of muscle. EMBO Rep.

[B3] Snider L, Tapscott SJ (2003). Emerging parallels in the generation and regeneration of skeletal muscle. Cell.

[B4] Huh MS, Smid JK, Rudnicki MA (2005). Muscle function and dysfunction in health and disease. Birth Defects Res C Embryo Today.

[B5] Mayer U (2003). Integrins: redundant or important players in skeletal muscle?. J Biol Chem.

[B6] Brzoska E, Bello V, Darribere T, Moraczewski J (2006). Integrin alpha3 subunit participates in myoblast adhesion and fusion in vitro. Differentiation.

[B7] Schwander M, Leu M, Stumm M, Dorchies OM, Ruegg UT, Schittny J, Muller U (2003). Beta1 integrins regulate myoblast fusion and sarcomere assembly. Dev Cell.

[B8] Guo C, Willem M, Werner A, Raivich G, Emerson M, Neyses L, Mayer U (2006). Absence of alpha 7 integrin in dystrophin-deficient mice causes a myopathy similar to Duchenne muscular dystrophy. Hum Mol Genet.

[B9] Hayashi YK, Chou FL, Engvall E, Ogawa M, Matsuda C, Hirabayashi S, Yokochi K, Ziober BL, Kramer RH, Kaufman SJ (1998). Mutations in the integrin alpha7 gene cause congenital myopathy. Nat Genet.

[B10] Lo SH (2006). Focal adhesions: what's new inside. Dev Biol.

[B11] Gheyara AL, Vallejo-Illarramendi A, Zang K, Mei L, St-Arnaud R, Dedhar S, Reichardt LF (2007). Deletion of integrin-linked kinase from skeletal muscles of mice resembles muscular dystrophy due to alpha 7 beta 1-integrin deficiency. Am J Pathol.

[B12] Huang Y, Li J, Zhang Y, Wu C (2000). The roles of integrin-linked kinase in the regulation of myogenic differentiation. J Cell Biol.

[B13] Dalkilic I, Schienda J, Thompson TG, Kunkel LM (2006). Loss of FilaminC (FLNc) results in severe defects in myogenesis and myotube structure. Mol Cell Biol.

[B14] Quach NL, Rando TA (2006). Focal adhesion kinase is essential for costamerogenesis in cultured skeletal muscle cells. Dev Biol.

[B15] Shi X, Ma YQ, Tu Y, Chen K, Wu S, Fukuda K, Qin J, Plow EF, Wu C (2007). The MIG-2/integrin interaction strengthens cell-matrix adhesion and modulates cell motility. J Biol Chem.

[B16] Tu Y, Wu S, Shi X, Chen K, Wu C (2003). Migfilin and Mig-2 link focal adhesions to filamin and the actin cytoskeleton and function in cell shape modulation. Cell.

[B17] Ussar S, Wang HV, Linder S, Fassler R, Moser M (2006). The Kindlins: subcellular localization and expression during murine development. Exp Cell Res.

[B18] Siegel DH, Ashton GH, Penagos HG, Lee JV, Feiler HS, Wilhelmsen KC, South AP, Smith FJ, Prescott AR, Wessagowit V (2003). Loss of kindlin-1, a human homolog of the Caenorhabditis elegans actin-extracellular-matrix linker protein UNC-112, causes Kindler syndrome. Am J Hum Genet.

[B19] Rogalski TM, Mullen GP, Gilbert MM, Williams BD, Moerman DG (2000). The UNC-112 gene in Caenorhabditis elegans encodes a novel component of cell-matrix adhesion structures required for integrin localization in the muscle cell membrane. J Cell Biol.

[B20] Dowling JJ, Gibbs E, Russell M, Goldman D, Minarcik J, Golden JA, Feldman EL (2008). Kindlin-2 is an essential component of intercalated discs and is required for vertebrate cardiac structure and function. Circ Res.

[B21] Ervasti JM (2003). Costameres: the Achilles' heel of Herculean muscle. J Biol Chem.

[B22] Ohtake Y, Tojo H, Seiki M (2006). Multifunctional roles of MT1-MMP in myofiber formation and morphostatic maintenance of skeletal muscle. J Cell Sci.

[B23] Steffensen B, Magnuson VL, Potempa CL, Chen D, Klebe RJ (1992). Alpha 5 integrin subunit expression changes during myogenesis. Biochim Biophys Acta.

[B24] George-Weinstein M, Foster RF, Gerhart JV, Kaufman SJ (1993). *In vitro* and *in vivo* expression of alpha 7 integrin and desmin define the primary and secondary myogenic lineages. Dev Biol.

[B25] Mackinnon AC, Qadota H, Norman KR, Moerman DG, Williams BD (2002). C. elegans PAT-4/ILK functions as an adaptor protein within integrin adhesion complexes. Curr Biol.

[B26] McGrath MJ, Mitchell CA, Coghill ID, Robinson PA, Brown S (2003). Skeletal muscle LIM protein 1 (SLIM1/FHL1) induces alpha 5 beta 1-integrin-dependent myocyte elongation. Am J Physiol Cell Physiol.

[B27] White DE, Coutu P, Shi YF, Tardif JC, Nattel S, St Arnaud R, Dedhar S, Muller WJ (2006). Targeted ablation of ILK from the murine heart results in dilated cardiomyopathy and spontaneous heart failure. Genes Dev.

[B28] Legate KR, Montanez E, Kudlacek O, Fassler R (2006). ILK, PINCH and parvin: the tIPP of integrin signalling. Nat Rev Mol Cell Biol.

[B29] Wu C (2005). PINCH, N(i)ck and the ILK: network wiring at cell-matrix adhesions. Trends Cell Biol.

[B30] Lu H, Fedak PW, Dai X, Du C, Zhou YQ, Henkelman M, Mongroo PS, Lau A, Yamabi H, Hinek A (2006). Integrin-linked kinase expression is elevated in human cardiac hypertrophy and induces hypertrophy in transgenic mice. Circulation.

[B31] Knoll R, Postel R, Wang J, Kratzner R, Hennecke G, Vacaru AM, Vakeel P, Schubert C, Murthy K, Rana BK (2007). Laminin-alpha4 and integrin-linked kinase mutations cause human cardiomyopathy via simultaneous defects in cardiomyocytes and endothelial cells. Circulation.

[B32] Hannigan GE, Coles JG, Dedhar S (2007). Integrin-linked kinase at the heart of cardiac contractility, repair, and disease. Circ Res.

[B33] Windpassinger C, Schoser B, Straub V, Hochmeister S, Noor A, Lohberger B, Farra N, Petek E, Schwarzbraun T, Ofner L (2008). An X-linked myopathy with postural muscle atrophy and generalized hypertrophy, termed XMPMA, is caused by mutations in FHL1. Am J Hum Genet.

[B34] Quinzii CM, Vu TH, Min KC, Tanji K, Barral S, Grewal RP, Kattah A, Camano P, Otaegui D, Kunimatsu T (2008). X-linked dominant scapuloperoneal myopathy is due to a mutation in the gene encoding four-and-a-half-LIM protein 1. Am J Hum Genet.

